# A Novel Prognostic Scoring System Using Inflammatory Response Biomarkers for Esophageal Squamous Cell Carcinoma

**DOI:** 10.1007/s00268-017-4144-y

**Published:** 2017-07-25

**Authors:** Noriyuki Hirahara, Yoshitsugu Tajima, Yusuke Fujii, Tetsu Yamamoto, Ryoji Hyakudomi, Takanori Hirayama, Takahito Taniura, Kazunari Ishitobi, Akihiko Kidani, Yasunari Kawabata

**Affiliations:** Department of Digestive and General Surgery, Faculty of Medicine, Shimane University, 89-1 Enya-cho, Izumo, Shimane 693-8501 Japan

## Abstract

**Background:**

We describe a novel scoring system, namely the inflammatory response biomarker (IRB) score. The aim of this study is to evaluate the clinical value of IRB score in patients undergoing curative resection for esophageal squamous cell carcinoma (SCC).

**Methods:**

We retrospectively reviewed patients who underwent curative esophagectomy. We evaluated IRB score in both non-elderly (<70 years) and elderly (≥70 years) SCC patients. The IRB score was determined as follows: a high lymphocyte-to-monocyte ratio (LMR) (>4), a high neutrophil-to-lymphocyte ratio (NLR) (>1.6), and a low platelet-to-lymphocyte ratio (PLR) (<147) were each scored as 1, and the remaining values were scored as 0; the individual scores were then summed to produce the IRB score (range 0−3).

**Results:**

Univariate analyses demonstrated that the TNM pStage (*p* < 0.0001), tumor size (*p* = 0.002), LMR (*p* = 0.0057), PLR (*p* = 0.0328) and IRB score (*p* = 0.0003) were significant risk factors for a worse prognosis. On multivariate analysis, the TNM pStage (*p* < 0.0001) and IRB score (*p* = 0.0227) were independently associated with worse prognosis in overall patients. Among non-elderly patients, multivariate analyses demonstrated that the pStage (*p* = 0.0015) and IRB score (*p* = 0.0356) were independent risk factors for a worse prognosis. Among elderly patients, multivariate analysis demonstrated that the pStage (*p* = 0.0016), and IRB score (*p* = 0.0102) were independent risk factors for a worse prognosis.

**Conclusion:**

The present study provides evidence that the preoperative IRB score can be considered a promising independent prognostic factor of cancer-specific survival in patients undergoing curative resection for SCC, and that its predictive ability is useful in both non-elderly and elderly patients.

## Introduction

Esophageal carcinoma is a highly aggressive disease with poor prognosis and is usually fatal. It is estimated that it is the eighth most common cancer and fourth leading cause of cancer-related mortality worldwide [1]. Recent investigations have shown that cancer-related inflammation leads to worse prognosis. It is clear that the host’s inflammatory response to the tumor plays a key role in cancer development, progression, and metastasis [2]. Based on this knowledge, a number of inflammation-based prognostic parameters such as the lymphocyte-to-monocyte ratio (LMR), neutrophil-to-lymphocyte ratio (NLR), and platelet-to-lymphocyte ratio (PLR) have been investigated in several types of cancers. In particular, low LMR, low NLR, and high PLR are each known to be strong predictors of postoperative survival in several types of cancers [3–5]. These parameters are routinely measured by automated hematology analyzers in daily medical practice; they are easily available and inexpensive, which is one of the major advantages of their clinical application. However, their prognostic significance in esophageal cancer is yet to be determined.

With the steady increase in average life expectancy due to advances in medical sciences, esophageal squamous cell carcinoma (SCC) rates have been increasing worldwide, especially in elderly people [6]. Treatments for esophageal SCC include surgery, radiation, chemotherapy, or a combination thereof [7]. Although esophagectomy has now become the treatment of choice (even for elderly patients), and perioperative management strategies have improved, both open and thoracoscopic esophagectomies are considerably invasive [8]. Chemotherapy is also an important treatment component for esophageal SCC; however, not all elderly patients can tolerate the planned cycles of adjuvant chemotherapy to completion. Indeed, many of these patients suspend chemotherapy for various reasons, including drug toxicity [9]. Therefore, it is important to identify patients who are most at risk of developing postoperative recurrence in order to better customize management strategies according to the risk of recurrence.

In this study, we evaluated a novel prognostic scoring system that utilizes the LMR, NLR, and PLR, namely the inflammatory response biomarker (IRB) score, in esophageal SCC patients.

## Materials and methods

### Patients

**Fig. 1:**
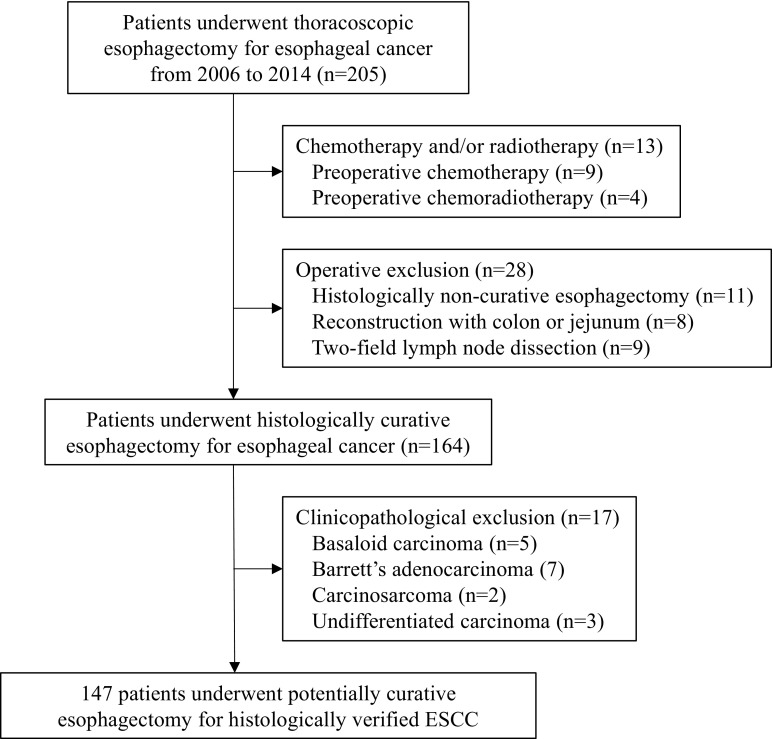
Flow diagram of this study

We retrospectively reviewed a database of 147 consecutive patients who underwent potentially curative esophagectomy with R0 resection for histologically verified esophageal SCC at our institute between January 2006 and December 2014. R0 resection was defined as a complete resection of the tumor with no microscopic margin involvement. During the study period, 205 patients received thoracoscopic esophagectomy for esophageal cancer. Of these, 13 patients received chemotherapy and/or radiotherapy, and 28 patients excluded operative factors. And 17 patients exclude clinicopathological factors (Fig. 1). Video-assisted or thoracoscopic esophagectomy with three-field lymph node dissection was performed for all patients, followed by elevation of the gastric conduit to the neck via the posterior mediastinal approach or retrosternal approach with end-to-end anastomosis of the cervical esophagus and gastric conduit. The patients’ clinical characteristics, laboratory data, treatment, and pathological data were obtained from their medical records. No patients had clinical signs of infection, preoperatively. But in the study population, there is no data on the medication situation of nonsteroidal anti-inflammatory drugs (NSAIDs). Furthermore, we excluded patients who had received pre- or postoperative adjuvant chemotherapy and/or radiotherapy. On the other side, in all study population, 46 patients had relapsed and received chemotherapy, such as S-1 or 5-fluorouracil, and cisplatin was administered in all patients with recurrent and/or metastatic ESCC.

The observation period began on the day of surgery and continued for 5 years or until death, loss to follow-up, or withdrawal of consent. The cause of death was determined from case notes or computerized records, and the cancer-specific survival (CSS) was calculated. Two patients who died of complications related to surgery within 60 days after esophagectomy were excluded from the analysis. We defined ‘elderly’ patients as those aged 70 years or older and ‘non-elderly’ as those under 70 years (Pohl et al, 2005). This retrospective study was registered with the ethical board of our institution and was conducted in accordance with the Declaration of Helsinki.

### Blood sample analysis

Preoperative complete blood cell (CBC) counts were retrospectively extracted from the patients’ medical records. Only patients with available preoperative CBC counts and blood differential data were included in the study. All white blood cell counts and differentials were obtained within 1 week before surgery. CBCs obtained from ethylenediaminetetraacetic acid-treated blood were analyzed by using an automated hematology analyzer XE-5000 (Medical Electronics, Kobe, Japan). The absolute counts of lymphocytes, monocytes, and platelets were obtained from the CBC data.

### Calculation of LMR, NLR, and PLR

The LMR was calculated by dividing the absolute lymphocyte count by the absolute monocyte count as obtained during a routine preoperative blood count. White blood cell counts were measured at the general testing laboratory at our hospital; the NLR was calculated by dividing the number of absolute neutrophils by the number of absolute lymphocytes according to the white blood cell differential. The PLR was calculated by dividing the absolute platelet count by the absolute lymphocyte count.

The optimal cutoff levels of the LMR, NLR, and PLR were determined via receiver operating curve (ROC) analysis. For the LMR, the area under the curve (AUC) and cutoff level for predicting CSS were 0.69 and 4.0, respectively, with a sensitivity of 62.5% and a specificity of 71.3%. For the NLR, the AUC and cutoff level for CSS prediction were 0.58 and 1.6, respectively, with a sensitivity of 57.5% and a specificity of 66.3%. For the PLR, the AUC and cutoff level for predicting CSS were 0.65 and 147, respectively, with a sensitivity of 59.6% and a specificity of 68.4%. Values above the cutoffs were considered high.

### Calculation of IRB scores

The IRB score was determined as follows: a high LMR (>4), a high NLR (>1.6), and a low PLR (<147) were each scored as 1, and the remaining values were scored as 0; the individual scores were then summed to produce the IRB score (range 0−3) [10].

### Staging

The pathological classification of the primary tumor, degree of lymph node involvement, and presence of organ metastasis were determined according to the TNM classification system [11].

### Statistical analysis

Means and standard deviations were calculated, and differences between the study groups were evaluated by using Student’s *t* test. Differences between the clinicopathological features were analyzed by using the Chi-square test. CSS was calculated via Kaplan–Meier analysis, and the differences between the groups were assessed by using the log-rank test. Prognostic factors associated with decreased survival rates were identified via Cox regression analysis.

Univariate analyses were performed to identify the variables associated with CSS. Variables with a *p*-value < 0.05 on univariate analyses were subjected to multivariate logistic regression analysis. The potential prognostic factors for esophageal cancer were as follows: age (<70 vs. ≥70 years); sex; TNM pathologic stage (I and II vs. III); tumor size (<3 cm vs. ≥3 cm); operation time (<600 min vs. ≥600 min); intraoperative blood loss (<500 mL vs. ≥500 mL); LMR (<4 vs. ≥4); NLR (<1.6 vs. ≥1.6); PLR (<147 vs. ≥147); serum SCC antigen level (<1.5 ng/ml vs. ≥1.5 ng/ml); and IRB score (2 or 3 vs. 0 or 1). Medical records were retrospectively reviewed to obtain all necessary data.

All statistical analyses were performed by using the JMP software (version 11 for Windows; SAS Institute, Cary, NC, USA); *p*-values <0.05 were considered statistically significant.

## Results

### Associations between the LMR, NLR, and PLR and clinicopathological features in patients with esophageal SCC

The relationships between the inflammatory response parameters (LMR, NLR, and PLR) and the clinicopathological features of 147 patients with esophageal SCC are shown in Table 1. The LMR significantly correlated with the lymphocyte count (*p* < 0.0001), monocyte count (*p* < 0.0001), tumor size (*p* = 0.014), tumor depth (*p* = 0.0004), and TNM pathologic stage (*p* = 0.0002). The NLR significantly correlated with the white blood cell count (*p* = 0.016), neutrophil count (*p* < 0.0001), lymphocyte count (*p* < 0.0001), and tumor depth (*p* = 0.002). The PLR significantly correlated with the lymphocyte count (*p* < 0.0001), platelet count (*p* < 0.0001), and tumor location (*p* = 0.042). It was notable that the LMR significantly correlated with more advanced TNM pathologic stages, while the NLR and PLR did not.

**Table 1:** Relationships between LMR, NLR, PLR and clinicopathological features in 147 patients with esophageal cancer

Characteristics	Total patients	LMR			NLR			PLR		
		<4	≥4		1.6<	≥1.6		147<	≥147	
		(*n*=65)	(*n*=82)	*p* value	(*n*=37)	(*n*=110)	*p* value	(*n*=79)	(*n*=68)	*p* value
Age (years)		65.8 ± 7.4	65.7 ± 8.2	0.934	65.4 ± 8.0	65.9 ± 7.9	0.72	66.8 ± 8.1	64.6 ± 7.6	0.097
Sex				0.052			0.163			0.562
Male	132	62	70		31	101		72	60	
Female	15	3	12		6	9		7	8	
WBC		6082.2 ± 2153.2	5844.3 ± 1788.2	0.466	5284.1 ± 1667.3	6171.2 ± 1996.5	0.016	6190.9 ± 1723.0	5665.6 ± 2167.2	0.104
Neutrophil		3944.7 ± 1804.6	3412.8 ± 1470.4	0.051	2491.0 ± 948.3	4032.3 ± 1643.7	<0.0001	3509.3 ± 1300.5	3801.3 ± 1960.9	0.283
Lymphocyte		1322.0 ± 546.4	1942.5 ± 584.5	<0.0001	2187.6 ± 658.6	1499.0 ± 541.8	<0.0001	2029.2 ± 586.3	1257.7 ± 426.2	<0.0001
Monocyte		546.8 ± 211.3	328.7 ± 111.1	<0.0001	379.0 ± 161.3	438.7 ± 203.3	0.1074	418.2 ± 171.3	430.0 ± 220.2	0.714
Platelet		236.6 ± 79.2	226.9 ± 66.2	0.42	231.0 ± 76.9	231.2 ± 70.7	0.987	203.5 ± 49.2	263.2 ± 80.9	<0.0001
Location of tumor				0.087			0.313			0.042
Ce	6	5	1		1	5		0	6	
Ut	8	4	4		0	8		5	3	
Mt	65	29	36		20	45		32	33	
Lt	52	24	28		11	41		31	21	
Ae	16	3	13		5	11		11	5	
Tumor size (mm)		4.9 ± 1.9	3.9 ± 2.7	0.014	3.8 ± 2.8	4.5 ± 2.3	0.134	4.0 ± 2.5	4.8 ± 2.3	0.056
Depth of tumor				0.0004			0.002			0.06
T1a–1b	66	20	46		18	48		40	26	
2	12	2	10		8	4		9	3	
3	56	34	22		8	48		26	30	
4a–4b	13	9	4		3	10		4	9	
Lymph node metastasis				0.198			0.1532			0.0639
N0	79	30	49		22	57		43	36	
N1	42	19	23		12	30		25	17	
N2	12	8	4		3	9		8	4	
N3	14	8	6		0	14		3	11	
Pathological stage				0.0002			0.1338			0.3497
1a–1b	59	14	45		20	39		36	23	
2a–2b	33	21	12		6	27		16	17	
3a–3c	55	30	25		11	44		27	28	
Operation time (min)		644.8 ± 162.2	663.5 ± 159.2	0.4843	655.9 ± 177.2	655.2 ± 155.0	0.9798	676.5 ± 149.0	630.8 ± 170.2	0.0845
Intraoperative blood loss (ml)		751.8 ± 622.8	581.6 ± 633.4	0.1059	568.8 ± 511.1	684.9 ± 667.8	0.3359	598.5 ± 633.1	722.2 ± 629.7	0.2384
SCC antigen		1.19 ± 1.06	1.12 ± 1.12	0.7208	1.04 ± 1.12	1.19 ± 1.08	0.7643	1.05 ± 0.91	1.27 ± 1.26	0.8858

### Associations between the IRB score and clinicopathological features in patients with esophageal SCC

The relationships between the IRB score and clinicopathological features of the 147 patients with esophageal SCC are shown in Table 2. Since the number of patients with an IRB score of 0 was small, we combined patients with IRB scores of 0 and 1 into a single category and compared then to those with IRB scores of 2 and 3.

**Table 2:** Relationships between IRB score and clinicopathological features in 147 patients with esophageal

Cancer characteristics	Total patients	IRB 0–1 (0: *n*=3) (1: *n*=51)	IRB2 (*n*=61)	IRB 3 (*n*=32)	*p* value
Age (years)		65.6 ± 7.4	64.5 ± 8.0	68.9 ± 8.1	0.0337
Sex					0.912
Male	132	49	54	29	
Female	15	5	7	3	
WBC		5627.6 ± 2183.5	6041.6 ± 1810.0	6309.7 ± 1767.4	0.2616
Neutrophil		3655.6 ± 1929.2	3506.1 ± 1552.1	3889.0 ± 1241.1	0.5664
Lymphocyte		1259.5 ± 537.4	1901.2 ± 624.7	1932.7 ± 490.0	<0.0001
Monocyte		485.2 ± 232.6	414.4 ± 174.1	337.4 ± 114.8	0.0023
Platelet		258.5 ± 79.4	220.0 ± 69.6	206.3 ± 46.1	0.0012
Location of tumor					0.0074
Ce	6	6	0	0	
Ut	8	2	3	3	
Mt	65	28	26	11	
Lt	52	17	21	14	
Ae	16	1	11	4	
Tumor size (mm)		5.0 ± 2.1	3.9 ± 2.6	4.0 ± 2.5	0.0335
Depth of tumor					0.0218
T1a–1b	66	17	30	19	
2	12	3	8	1	
3	56	27	17	12	
4a–4b	13	7	6	0	
Lymph node metastasis					0.3798
N0	79	28	33	18	
N1	42	15	17	10	
N2	12	3	8	1	
N3	14	8	3	3	
Pathological stage					0.341
1a–1b	59	16	27	16	
2a–2b	33	15	13	5	
3a–3c	55	23	21	11	
Operation time (min)		628.4 ± 179.3	655.8 ± 147.6	700.2 ± 143.1	0.1332
Intraoperative blood loss (ml)		731.5 ± 582.0	632.0 ± 642.7	572.8 ± 697.6	0.4969
SCC antigen		1.23 ± 1.09	1.14 ± 1.28	1.05 ± 0.60	0.7669
LMR					<0.0001
<4	65	46	19	0	
≥4	82	8	42	32	
NLR					0.0005
1.6<	40	14	25	1	
≥1.6	107	40	36	31	
PLR					<0.0001
147<	79	3	44	32	
≥147	68	51	17	0	

The IRB score significantly correlated with age (*p* = 0.0337), lymphocyte count (*p* < 0.0001), monocyte count (*p* = 0.0023), platelet count (*p* = 0.0012), tumor location (*p* = 0.0074), tumor size (*p* = 0.0335), tumor depth (*p* = 0.0218), LMR (*p* < 0.0001), NLR (*p* = 0.0005), and PLR (*p* < 0.0001). However, there was no significant association between IRB score and TNM pathologic stage.

### Associations between the IRB score and clinicopathological features in non-elderly patients

Associations between the IRB score and clinicopathological features in 91 patients younger than 70 years (the non-elderly group) are shown in Table 3.

**Table 3:** Relationships between IRB score and clinicopathological features in 91 non-elderly patients with esophageal cancer

Characteristics	Total patients	IRB 0–1 (*n*=34)	IRB 2 (*n*=39)	IRB 3 (*n*=18)	*p* value
Sex					0.7158
Male	83	30	36	17	
Female	8	4	3	1	
WBC		5924.4 ± 2324.7	6169.5 ± 2031.2	6281.7 ± 1531.7	0.8071
Neutrophil		3837.7 ± 1929.2	3634.9 ± 1753.2	3934.3 ± 1143.0	0.797
Lymphocyte		1303.2 ± 637.7	1924.9 ± 683.6	1887.2 ± 370.3	<0.0001
Monocyte		522.5 ± 254.8	417.9 ± 173.1	322.9 ± 90.3	0.0026
Platelet		255.2 ± 80.3	227.6 ± 78.5	212.4 ± 40.4	0.1021
Location of tumor					0.0312
Ce	4	4		0	0
Ut	2	1		0	1
Mt	42	20		16	6
Lt	31	8		16	7
Ae	12	1		7	4
Tumor size (mm)		4.8 ± 1.9	4.0 ± 2.8	4.1 ± 3.1	0.4544
Depth of tumor					0.0286
T1a–1b	40	8		21	11
2	6	2		4	0
3	36	19		10	7
4a–4b	9	5		4	0
Lymph node metastasis					0.6385
N0	51	18		24	9
N1	24	10		8	6
N2	6	2		4	0
N3	10	4		3	3
Pathological stage					0.1811
1a–1b	38	9		20	9
2a–2b	18	10		6	2
3a–3c	35	15		13	7
Operation time (min)		587.0 ± 142.9	660.8 ± 141.6	721.5 ± 151.3	0.0054
Intraoperative blood loss (ml)		736.5 ± 588.0	579.4 ± 556.6	494.4 ± 488.7	0.2758
SCC antigen		1.07 ± 0.79	1.13 ± 1.26	1.06 ± 0.64	0.9577
LMR					<0.0001
<4	40	29	11	0	
≥4	51	5	28	18	
NLR					0.0288
1.6<	24	8	15	1	
≥1.6	67	26	24	17	
PLR					<0.0001
147<	47	3	26	18	
≥147	44	31	13	0	

The IRB score significantly correlated with the lymphocyte count (*p* < 0.0001), monocyte count (*p* = 0.0026), tumor location (*p* = 0.0312), depth of tumor (*p* = 0.0286), surgery time (*p* = 0.0054), LMR (*p* < 0.0001), NLR (*p* = 0.0288), and PLR (*p* < 0.0001). However, there was no significant association between IRB score and TNM pathologic stage.

### Associations between the IRB score and clinicopathological features in elderly patients

Associations between the IRB score and clinicopathological features in patients 70 years of age or older (the elderly group; *n* = 56) are shown in Table 4. The IRB score significantly correlated with the lymphocyte count (*p* < 0.0001), platelet count (*p* = 0.0041), tumor size (*p* = 0.0179), LMR (*p* < 0.0001), NLR (*p* = 0.013), and PLR (*p* < 0.0001). However, there was no significant association between the IRB score and TNM pathologic stage.

**Table 4:** Relationships between IRB score and clinicopathological features in 56 elderly patients with esophageal cancer

Characteristics	Total patients	IRB 0–1 (*n*=20)	IRB2 (*n*=22)	IRB 3 (*n*=14)	*p* value
Sex					0.4234
Male	49	19	18	12	
Female	7	1	4	2	
WBC		5123.0 ± 1867.3	5815.0 ± 1345.4	6345.7 ± 2092.4	0.1322
Neutrophil		3346.0 ± 1938.4	3277.8 ± 1111.5	3830.7 ± 1400.0	0.5396
Lymphocyte		1185.4 ± 300.2	1859.1 ± 516.3	1991.1 ± 621.9	<0.0001
Monocyte		421.7 ± 177.2	408.3 ± 179.6	356.1 ± 141.8	0.5239
Platelet		264.0 ± 79.8	206.4 ± 48.8	198.6 ± 53.0	0.0041
Location of tumor					0.1083
Ce	2	2	0	0	
Ut	6	1	3	2	
Mt	23	8	10	5	
Lt	21	9	5	7	
Ae	4	0	4	0	
Tumor size (mm)		5.4 ± 2.3	3.7 ± 2.1	3.8 ± 1.6	0.0179
Depth of tumor					0.6818
T1a–1b	26	9	9	8	
2	6	1	4	1	
3	20	8	7	5	
4a–4b	4	2	2	0	
Lymph node metastasis					0.0898
N0	51	10	9	9	
N1	24	5	9	4	
N2	6	1	4	1	
N3	10	4	0	0	
Pathological stage					0.8278
1a–1b	21	7	7	7	
2a–2b	15	5	7	3	
3a–3c	20	8	8	4	
Operation time (min)		698.8 ± 214.3	646.7 ± 160.7	672.7 ± 132.0	0.635
Intraoperative blood loss (ml)		723.1 ± 586.8	725.5 ± 778.1	673.6 ± 910.3	0.9764
SCC antigen		1.49 ± 1.45	1.15 ± 1.35	1.04 ± 0.56	0.5334
LMR					<0.0001
<4	25	17	8	0	
≥4	31	3	14	14	
NLR					0.0130
1.6<	16	6	10	0	
≥1.6	40	14	12	14	
PLR					<0.0001
147<	32	0	18	14	
≥147	24	20	4	0	

### Prognostic factors for CSS in overall patients with esophageal SCC

Univariate analyses demonstrated that the TNM pStage (*p* < 0.0001), tumor size (*p* = 0.002), LMR (*p* = 0.0057), PLR (*p* = 0.0328), and IRB score (*p* = 0.0003) were significant risk factors for a worse prognosis (Table 5). On multivariate analysis, the TNM pStage (*p* < 0.0001) and IRB score (*p* = 0.0227) were independently associated with worse prognosis (Table 5).

**Table 5:** Univariate and multivariate analyses to assess the prognostic factors for overall esophageal cancer

Variables	Patients (*n*=147)	Category or characteristics	Univariate			Multivariate		
			HR	95% CI	*p* value	HR	95% CI	*p* value
Sex	15/132	(Female/male)	1.031	0.447–2.990	0.9487			
Age	56/91	(70</≥70)	1.089	0.586–1.961	0.7819			
pStage	92/55	(1, 2/3)	4.938	2.711–9.330	<0.0001	4.47	2.374–8.768	<0.0001
Tumor size	45/102	(3</≥3)	3.086	1.469–7.545	0.002	1.495	0.638–3.915	0.3659
Operation time	99/48	(600</≥600)	1.792	0.996–3.206	0.0514			
Intraoperative blood loss	72/75	(500</≥500)	1.239	0.693–2.250	0.4706			
LMR	82/65	(≥4.0/4.0<)	2.279	1.272–4.169	0.0057	1.125	0.442–2.965	0.8088
NLR	37/110	(≥1.6/1.6<)	1.291	0.678–2.354	0.4232			
PLR	79/68	(147</≥147)	1.886	1.053–3.444	0.0328	1.691	0.633–4.619	0.3009
SCC	109/38	(1.5</≥1.5)	1.555	0.767–2.947	0.2102			
IRB score	54/93	(0, 1/2, 3)	2.918	1.630–5.301	0.0003	4.271	1.219–15.743	0.0227

### Prognostic factors for CSS in non-elderly patients with esophageal SCC

Among non-elderly patients, univariate analyses showed that the TNM pStage (*p* < 0.0001), tumor size (*p* = 0.001), LMR (*p* = 0.0045), PLR (*p* = 0.0439), and IRB score (*p* = 0.0021) were significantly associated with a worse prognosis (Table 6). Multivariate analyses demonstrated that pStage (*p* = 0.0015), and IRB score (*p* = 0.0356) were independent risk factors for a worse prognosis in this group of patients (Table 6).

**Table 6:** Univariate and multivariate analyses to assess the prognostic factors for non-elderly patients with esophageal cancer

Variables	Patients (*n*=91)	Category or characteristics	Univariate			Multivariate		
			HR	95% CI	*p* value	HR	95% CI	*p* value
Sex	8/83	(Female/male)	0.487	0.186–1.664	0.2235			
pStage	56/35	(1, 2/3)	4.825	2.275–10.871	<0.0001	3.667	1.629–8.914	0.0015
Tumor size	29/62	(3</≥3)	5.169	1.818–21.687	0.001	2.208	0.682–10.458	0.1886
Operation time	60/31	(600</≥600)	1.968	0.940–4.140	0.0719			
Intraoperative blood loss	43/48	(500</≥500)	1.026	0.492–2.180	0.9455			
LMR	51/40	(≥4.0/4.0<)	2.937	1.393–6.587	0.0045	1.619	0.168–5.280	0.4407
NLR	24/67	(≥1.6/1.6<)	0.675	0.642–4.011	0.3752			
PLR	47/44	(147</≥147)	2.139	1.021–4.689	0.0439	1.409	0.437–4.509	0.5664
SCC	109/38	(1.5</≥1.5)	1.469	0.575–3.318	0.3973			
IRB score	34/57	(0, 1/2, 3)	3.05	1.461–6.571	0.0021	2.456	0.898–5.532	0.0356

### Prognostic factors for CSS in elderly patients with esophageal SCC

Among elderly patients, univariate analyses demonstrated that the TNM pStage (*p* = 0.0012), NLR (*p* = 0.049), and IRB score (*p* = 0.0158) were significantly associated with a worse prognosis (Table 7). Multivariate analysis demonstrated that the pStage (*p* = 0.0016), and IRB score (*p* = 0.0102) were independent risk factors for a worse prognosis in this group of patients (Table 7).

**Table 7:** Univariate and multivariate analyses to assess the prognostic factors for elderly patients with esophageal cancer

Variables	Patients (*n*=56)	Category or characteristics	Univariate			Multivariate		
			HR	95% CI	*p* value	HR	95% CI	*p* value
Sex	7/49	(Female/male)	3.671	0.740–66.526	0.1277			
pStage	36/20	(1, 2/3)	5.029	1.891–14.746	0.0012	5.758	2.108–17.472	0.0016
Tumor size	16/40	(3</≥3)	1.534	0.541–5.466	0.4398			
Operation time	39/17	(600</≥600)	1.547	0.561–4.033	0.3842			
Intraoperative blood loss	29/27	(500</≥500)	1.719	0.659–4.744	0.268			
LMR	31/25	(≥4.0/4.0<)	1.519	0.563–4.039	0.4001			
NLR	40/16	(≥1.6/1.6<)	3.194	1.217–8.535	0.049	3.995	1.448–11.399	0.0569
PLR	32/24	(147</≥147)	1.528	0.583–4.073	0.382			
SCC	109/38	(1.5</≥1.5)	1.644	0.518–4.493	0.373			
IRB score	20/36	(0, 1/2, 3)	2.704	1.019–7.327	0.0158	3.981	1.393–12.097	0.0102

### Postoperative CSS based on the LMR, NLR, and PLR in all patients with esophageal cancer

**Fig. 2:**
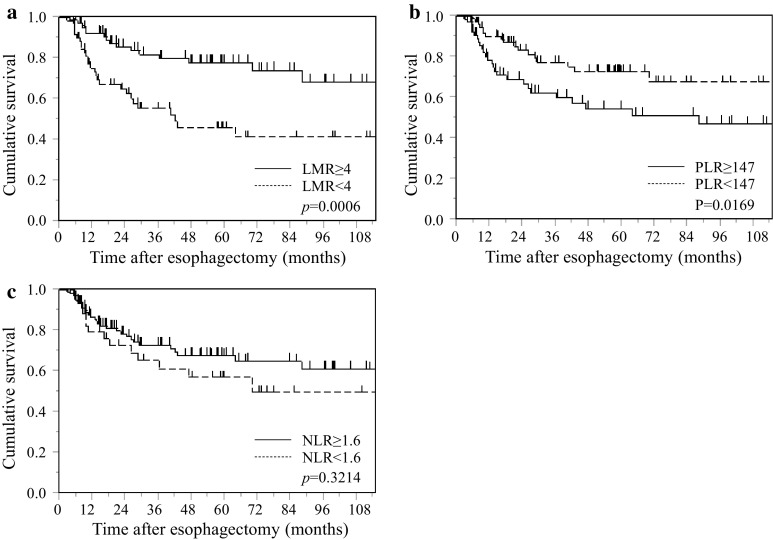
Kaplan–Meier survival curves showing the relationship between inflammatory response biomarkers and CSS after esophagectomy in overall patents with esophageal cancer. **a** LMR, **b** PLR, **c** NLR

Patients with a low LMR (*p* < 0.001; Fig. 2a) or a high PLR (*p* < 0.05; Fig. 2b) were associated with a significantly poorer CSS rate. Conversely, patients with a low NLR had a slight tendency toward poorer prognosis; however, the difference between the CSS rates was not significant (*p* = 0.321; Fig. 2c).

Postoperative CSS based on the LMR, NLR, and PLR in all patients with esophageal cancer.

### Postoperative CSS based on the IRB score in all patients with esophageal SCC

**Fig. 3:**
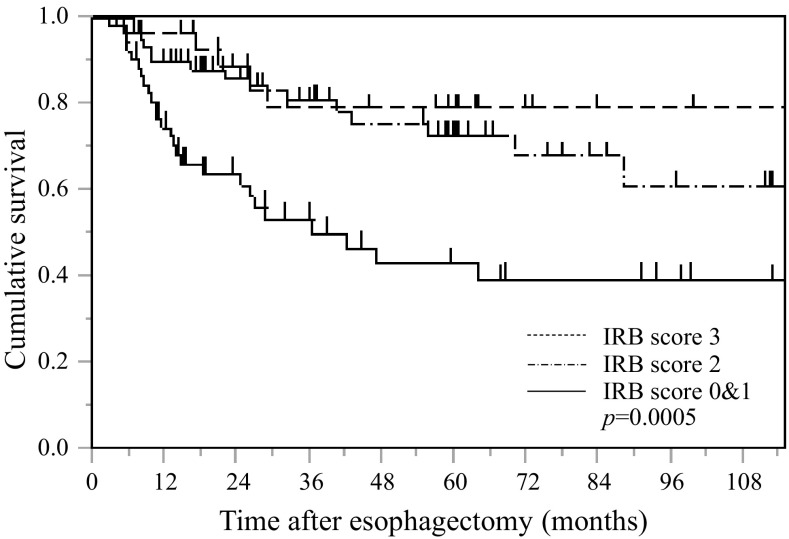
Kaplan–Meier survival curves showing the relationship between IRB score and CSS after esophagectomy in overall patents with esophageal cancer

Kaplan–Meier analysis and the log-rank test demonstrated a significant difference in CSS among the three IRB score groups (*p* = 0.0005) (Fig. 3). Patients with IRB scores of 0 or 1 had a worse prognosis than those with IRB scores of 2 and of 3. The 5-year survival rates for those with IRB scores of 0–1, 2, and 3 were 40.7, 78.7, and 81.3%, respectively.

### Postoperative CSS based on the IRB score in non-elderly patients with esophageal SCC

**Fig. 4:**
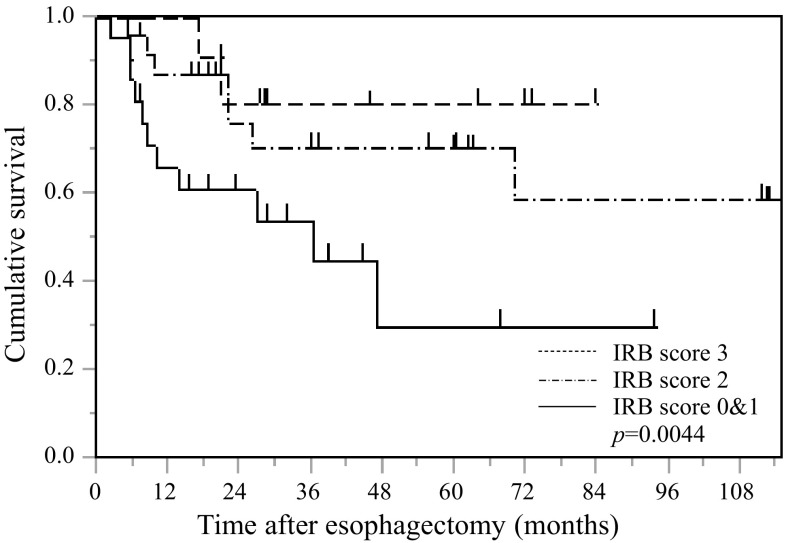
Kaplan–Meier survival *curves* showing the relationship between IRB score and CSS after esophagectomy in non-elderly patents with esophageal cancer

Kaplan–Meier analysis and the log-rank test demonstrated a significant difference in CSS among the three IRB score groups (*p* = 0.0044) (Fig. 4). Non-elderly patients with an IRB score of 0 or 1 had a worse prognosis than those with IRB scores of 2 or 3. The 5-year survival rates for patients with an IRB score of 0–1, 2, and 3 were 35.3, 77.0, and 77.8%, respectively.

### Postoperative CSS based on the IRB score in elderly patients with esophageal SCC

**Fig. 5:**
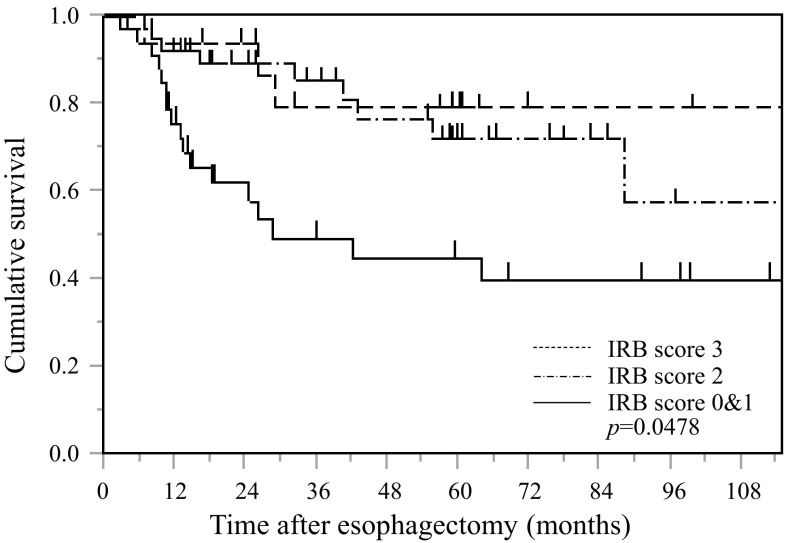
Kaplan–Meier survival *curves* showing the relationship between IRB score and CSS after esophagectomy in elderly patents with esophageal cancer

Kaplan–Meier analysis and the log-rank test demonstrated a significant difference in CSS among the three IRB score groups (*p* = 0.0478) (Fig. 5). Elderly patients with IRB scores of 0 or 1 had a worse prognosis than those with IRB scores of 2 and of 3. The 5-year survival rates for patients with IRB scores of 0–1, 2, and 3 were 45.0, 77.3, and 78.6%, respectively.

Taken together, these data showed that the preoperative IRB score was able to categorize esophageal SCC patients into three independent groups according to their anticipated prognosis after surgery.

## Discussion

With increasing evidence that the host’s SIRs are important prognostic indicators, a variety of prognostic biomarkers based on SIR have been described. Cancer-related inflammation leads to the suppression of antitumor immunity by recruiting regulatory T cells and activating chemokines, which in turn promote tumor growth and metastasis [12–14]. There is a strong link between inflammation and cancer. Since systemic chemotherapy or radiation will inevitably impact systemic inflammation significantly, evaluation of inflammation-based prognostic parameters such as LMR, NLR, and PLR, may not correctly reflect the baseline impact of systemic inflammation on survival in patients receiving neoadjuvant or adjuvant chemotherapy/chemoradiotherapy [15]. Therefore, we evaluated the potential prognostic role of preoperative IRB scores in esophagectomized SCC patients who had not received any pre- or postoperative adjuvant chemotherapy and/or radiotherapy.

In the past decade, a number of studies have confirmed the clinical utility of LMR, NLR, and PLR for predicting postoperative survival in patients with various types of solid tumors. However, their prognostic values and optimal cutoff points in esophageal cancer patients remained undetermined [16–18]. In a previous study, we generated ROC curves to determine the optimal cutoff values for predicting CSS in esophageal cancer patients, where the optimal cutoff values for the LMR, NLR, and PLR were 4.0, 1.6, and 147, respectively. We suggested that low LMR, low NLR, or high PLR independently predicts disease recurrence and shorter survival in esophageal SCC patients [2]. In this study, we designed the IRB scoring system, which combines the prognostic ability of the three parameters, and evaluated the ability of the IRB score to predict the survival of both elderly and non-elderly esophageal SCC patients after R0 resection.

The present study demonstrated that the LMR and PLR were significant prognostic factors in SCC patients on univariate analysis, but that they had no impact on survival on multivariate analysis. Meanwhile, multivariate analysis showed the IRB score to be an independent prognostic factor for CSS in both elderly and non-elderly patients. This was probably because the IRB scoring method enhanced the individual prognostic ability of each of LMR, NLR, and PLR by stratifying their predictive capabilities. Interestingly, the IRB showed no relation to pTNM stage, which was a separate independent prognostic factor for CSS on multivariate analysis. Additionally, the IRB score was unrelated to levels of tumor markers such as the SCC antigen. Because it is not unusual for patients with advanced esophageal SCC to have tumor marker levels within the normal range, postoperative surveillance using the IRB score instead of conventional tumor markers may benefit such patients. Because preoperative IRB score was identified as significant independent risk factors for CSS in ESCC patients, but not SCC antigen, in multivariate logistic regression analysis. Besides, the LMR and PLR were significant prognostic factors for patients overall and for non-elderly patients on univariate analysis, while the NLR was a significant prognostic factor among elderly patients; however, none of these factors were predictive on multivariate analysis.

Any relationship between these parameters and patient prognosis may be explained by interactions between the immune/inflammatory cells of the tumor and surrounding normal tissues that are important for cancer development and progression. Cancer patients may be in a state of chronic inflammation and immunosuppression, particularly those who are elderly [19]. In this study; however, we revealed that the IRB score was of predictive value in both non-elderly and elderly esophageal SCC patients, suggesting that it might encompass the prognostic values of each of its components and thus producing a combined predictive effect.

Previous studies showed that tumors produce tumor necrosis factor alpha, granulocyte colony-stimulating factor, interleukin-1 (IL-1), and IL-6, which may influence tumor-related SIR [20]. Theoretically, direct measurement of serum IL-6 levels is the optimal method to estimate SIR resulting from interactions between the tumor and the host tissue [21]. However, there are many unsolved problems associated with the routine measurement of IL-6 in cancer patients, including its high cost and inconvenience [22]. On the other hand, the IRB score is simple and reasonable to measure as a representative biomarker of SIR because of its low cost and convenience. Additionally, the IRB score may have greater applicability for estimating the SIR, because proliferation and differentiation of cellular components occur more rapidly after inflammatory cytokines are released, and the IRB score is calculated by three inflammatory markers, including the LMR, NLR, and PLR. Moreover, because repeated measurements of the IRB score can be performed with ease both before and after surgery, it can provide reliable data for the prediction of prognosis in patients with esophageal SCC. We found that the IRB score was capable of dividing esophageal cancer patients into three independent groups preoperatively according to their anticipated postoperative survival.

The limitations of our study include its retrospective nature, single-institution design, small sample size, and short follow-up durations. Moreover, we excluded patients who had undergone adjuvant chemotherapy and/or radiation therapy. Another limitation is that the biological mechanisms that explain the ability of systemic inflammatory factors to predict prognosis are yet to be elucidated. Thus, large prospective randomized controlled trials are required to confirm our preliminary findings. And, though hazard ratio of LMR and NLR was each different, I scored it with addition as simplicity. The coefficient of each inflammatory factor is calculated by analyzing more patients, and it will be possible for devising the more significant predictive scoring formula in future. Despite the above limitations; however, the present study provides evidence that the preoperative IRB score can be considered a promising independent prognostic factor of CSS in patients with resectable esophageal cancer, and that its predictive ability is useful in both non-elderly and elderly patients. Although IRB score in pre-operation could predict the postoperative prognosis in this study, it will be necessary to evaluate IRB score in both pre- and postoperation, in future. The IRB score is easy and inexpensive to determine and can potentially be used to help guide risk stratification and treatment decisions in patients with resectable esophageal cancer.
